# Comparing Attachment Networks During Middle Childhood in Two Contrasting Cultural Contexts

**DOI:** 10.3389/fpsyg.2018.01201

**Published:** 2018-07-17

**Authors:** Sophia D. Becke, Stephan Bongard

**Affiliations:** Department of Psychology, Goethe University Frankfurt, Frankfurt, Germany

**Keywords:** attachment figures, middle childhood, developmental task, cross-cultural comparison, ecocultural approach, attachment networks, Cameroon, Germany

## Abstract

Cultural psychology assumes that the ecocultural conditions of a particular setting shape children’s pathways, resulting in multiple adaptive solutions to universal developmental tasks. While the adaptivity of attachment and children’s psychosocial development during the early years has been thoroughly investigated, attachment research during middle childhood continues to reflect Western ideals of family. Adhering to ideas of monotropy, most studies only focus on parental attachment figures. However, this restricted empirical perspective does not only result in a Eurocentric bias, it also neglects theoretical reflections on the growing complexity of attachment during middle childhood, thus only considering a limited selection of all individuals contributing to the children’s feeling of security, even in Western settings. To investigate the variability and adaptivity of attachment during middle childhood, this study assessed children’s attachment figures in two extreme settings of development, introducing an exhaustive network perspective on attachment during this developmental stage. Children of the Cameroonian Nseh (*N* = 11) and German children from Bad Nauheim (*N* = 11) identified and differentiated all individuals contributing to their attachment need in an exploratory and transdisciplinary approach. The socio-structural composition of children’s attachment networks follows the context-specific systems of care and concepts of interconnectedness and the ecological features of each setting, resulting in marked differences between both contexts. The functional composition, however, reflects children’s preoccupation with similar developmental challenges across settings. Same-aged peers contribute to the children’s feeling of safety in both settings, thereby deviating from previous reflections on their subordinate relevance during middle childhood. Overall, these results support the adaptiveness of children’s attachment patterns while also demonstrating universal trends across contexts. They highlight the collective nature of attachment during middle childhood that exceeds the impact of individual dyads. Thus, broad and context-sensitive research strategies become a necessary addition to attachment research in order to generate an exhaustive understanding for children’s development across cultural contexts.

## Introduction

Childhood research in cultural psychology and cultural anthropology has established that early developmental settings result from context-specific ecocultural conditions (Whiting and Whiting, 1975; Levine, 2007; Montgomery, 2009). Thus, childhood development can take a multitude of possible pathways, each adapted to the specific challenges and resources of a particular environment and each setting finding a suitable solution for universal developmental tasks (Weisner, 2002; Greenfield et al., 2003). Consequently, the distinct context needs to become an inherent part of childhood research in order to adequately understand processes of psychosocial development in their adaptive significance (LeVine et al., 1994; Keller, 2014; LeVine, 2014).

The construct of attachment constitutes a common focal point when investigating psychological development. Attachment theory investigates children’s universal need for attachment, met by distinctive long-lasting bonds between the child and a caregiver. These attachment figures maintain children’s continuous feeling of security and comfort as a secure base and reestablish it during periods of distress as a safe haven. Guided by the concept of monotropy, attachment research has up to now mostly focused on mothers as the most important attachment figures (Bowlby, 1969; Ainsworth, 1989; Cassidy, 2016).

In the last years, attachment theory has been met with growing criticism. With attachment research attributing global universality to behavioral concepts developed based on Western ideals of childhood and on empirical data limited to Western samples, this criticism mostly refers to the neglect of contextual adaptiveness and the resulting Eurocentric perspective on childhood (LeVine, 2014; Weisner, 2014; Keller, 2016). Especially the concept of monotropy has been criticized for describing the limited reality of Western nuclear families compared to the globally more common broad social networks (Nsamenang, 1992; Arnett, 1995, 2008; Keller, 2016). Thus, cultural psychology has developed an alternative approach, guided by the assumption that attachment patterns, too, can take a multitude of pathways, shaped by ecocultural features of the distinct settings of development (Weisner, 2005; Keller, 2014).

There are two complementary approaches to investigate this adaptiveness of children’s developmental pathways and to make the ecocultural context an inherent part of this research: an ecologically informed research strategy, drafted specifically for attachment research, and the comparison of prototypical settings.

The ecologically informed approach to attachment highlights the context-specific variability of this aspect of children’s development and thus calls for an in-depth investigation into the selected developmental environment. This approach also urges the researcher to consider indigenous concepts of interconnectedness in order to investigate and understand patterns of attachment in their individual adaptiveness to a distinct ecocultural setting. Methodologically, this strategy stresses the importance of a transdisciplinary approach, introducing ethnographic data into attachment research (Weisner, 2005, 2014; LeVine and Norman, 2008; Keller, 2014, 2016; LeVine, 2014).

To investigate the variability and adaptiveness of childhood across developmental contexts, it is also common to compare prototypical settings, i.e., extreme developmental settings that exhibit a distinct variation of cultural and social concepts and practices, as well as ecological conditions, to contrast their impact (Kâğıtçıbaşı, 1996; Keller, 2007; Harkness et al., 2015). One of these approaches highlighting the range of developmental settings describes two prototypical sociodemographic communities, each representing extremes in their ideas on the relationship between the individual and the group, conceptualized with the two dimensions of autonomy and relatedness (Kâğıtçıbaşı, 1996; Keller et al., 2006; Keller, 2007). Resulting from the ecocultural environment, each setting has a distinct psychology that shapes the context-specific developmental paths of socialization, conveying context-specific competences and strategies of survival (Kâğıtçıbaşı, 1996; Greenfield et al., 2003; Keller, 2007, 2014).

The first prototype describes the developmental setting of traditional villages. These relational contexts are often illustrated using the Cameroonian Nso as an example (Keller, 2007). This environment is marked by low maternal education, a high number of children per mother, and an early onset of reproduction (Keller et al., 2006). The cultural model focuses on interdependence, considering the individual as heteronomous and interconnected with others, and values obedience, harmony in the hierarchically organized group and social responsibility (Nsamenang and Lamb, 1993; Kâğıtçıbaşı, 1996; Keller et al., 2006; Keller, 2007). Families in these small-scale communities live in large households and are integrated into extended social networks. The interconnectedness translates into a care system based on shared care (Keller, 2014), oftentimes also involving peers as caregivers (Weisner and Gallimore, 1977; Bryant, 1989). Children have an economic value, contributing to the mostly subsistence based economy from early on (Kâğıtçıbaşı, 1996; Keller, 2007). Early care focuses primarily on the infant’s health (Keller, 2007) and survival due to critical living conditions, with care peaking in the first months and years before being transferred to the next child (LeVine, 1974, 1988). The infant is constantly monitored and never left alone (Keller, 2007).

The second prototype describes middle-class families in autonomous Western contexts, often illustrated using samples from Berlin or Los Angeles (Keller, 2007). This setting is marked by high maternal education, few children per mother and a comparably late onset of reproduction (Keller et al., 2006; Keller, 2007). The cultural model of this context focuses on independence, perceiving the individual as self-contained (Kâğıtçıbaşı, 1996; Keller et al., 2006; Keller, 2007). Individuals in these large-scale postindustrial communities that are based on cash economy live in nuclear families (Kâğıtçıbaşı, 1996; Keller, 2007). Child care is primarily a dyadic and parental task, with mothers taking over the majority of practical responsibilities and only a few non-parental caregivers present, especially during the first years. Care strategies in this context convey values of self-maximization, assertiveness and initiative (Weisner, 1984, 2005; Keller, 2007, 2014). Early care focuses on communication and cognitive development (Keller, 2007), preparing the child for the challenges of school and the job market (LeVine, 1974, 1988). Children only hold psychological value due to the high affluence and public pension systems (Kâğıtçıbaşı, 1996; Keller, 2007). They learn to spend time alone from early on, reinforcing autonomy (Keller, 2007; Yovsi et al., 2009).

For the first years of development, the adaptiveness of socialization and attachment patterns has thoroughly been investigated, highlighting differences in children’s developmental pathways. It has been demonstrated that caregivers in autonomous settings support emotional expressiveness, socializing children toward self-expression and self-definition (Keller and Otto, 2009), with children explicitly expressing their distress in attachment situations (Ainsworth et al., 1978). In contrast, caregivers in relational contexts prefer calm and inexpressive children as investigated among the Cameroonian Nso. This inexpressiveness extends to children’s attachment behavior. When confronted with a stranger, a situation expected to be stressful according to attachment theory, they remain calm and relaxed, even on a physiological level. Children’s emotional regulation is promoted in this context since it allows for the introduction of several caregivers, lifting the mother’s burden and ensuring a constant supervision while integrating the child into an interconnected society (Otto, 2008, 2014; Keller and Otto, 2009).

However, for the later years of childhood, there is still little research on the adaptiveness of developmental pathways. Attachment research for this developmental stage continues to follow the concept of monotropy concerning children’s attachment figures. While the social world of children expands during middle childhood, it is assumed that parents continue to act as the most important attachment figures, thus being assorted to the top of a presumed hierarchy of relevance, attributed to the extended availability and stability of these ties (Kerns et al., 2005; Raikes and Thompson, 2005). However, it is also acknowledged that other individuals, peers and non-parental adults now hold attachment-relevant responsibilities. Yet, they are considered to only serve as temporary and most often context-specific substitutes (Ainsworth, 1989; Mayseless, 2005). While it is assumed that these relationships possess “attachment-related dynamics,” non-parental attachment figures are most often not recognized as full-value attachment figures (Mayseless, 2005, p. 7).

Despite the growing attention this developmental stage has gained in the last years, attachment research of middle childhood is still marked by three major research gaps: A cultural bias, a limited perspective, only investigating an excerpt of all attachment figures and methodological constraints, limiting the children’s own contributions.

Most attachment researchers of middle childhood have already expounded the problem that previously observed patterns could be culturally biased due to current samples and methodological approaches being limited to Western contexts (Kerns et al., 2006; Seibert and Kerns, 2009; Chen, 2015), thus acknowledging contextual adaptiveness of children’s attachment patterns. However, they have not yet begun to consequently include the specific developmental context of their Western samples in their studies in order to empirically reflect on the distinct processes of adaptation.

In addition to the cultural limitations of the available data on attachment during middle childhood, it is also the continuous adherence to a monotropic view that narrows the perspective of attachment research during this developmental stage. The resulting research approach is set on dyadic attachment ties to biological parents, especially mothers, and leads to the *a priori* subordination and extensive exclusion of peers and most non-parental adults from closer investigations. This does not only extend the Eurocentric bias to middle childhood, restricting the focus to nuclear, thus Western settings of childhood. Taking the expansion of children’s social environment and the development of new attachment ties during this developmental stage into consideration, in fact it also restricts research in Western contexts, reflecting only an excerpt of the growing attachment environment, with most studies focusing on the quality and impact of ties to top-tier attachment figures, thereby neglecting other levels of the assumed attachment hierarchy. As an alternative, a network perspective on attachment has been suggested, positioning the individual in their complete attachment environment (Lamb, 2005; Lewis, 2005). However, this approach has been disregarded by mainstream attachment research as of yet (Keller, 2016).

Finally, these studies on middle childhood are methodologically dominated by adult researchers and their own reflections on attachment, limiting the children’s own contribution (Becke and Bongard, 2018a) and restricting them to a subordinate position of rating preformulated statements on preselected attachment figures despite their growing ability to reflect on their attachment ties (Raikes and Thompson, 2005). Only one study thus far allowed children to freely identify the entirety of their attachment figures (Seibert and Kerns, 2009).

In order to develop an adequate understanding for the characteristics of the developmental task of attachment during middle childhood, it is necessary to explore the general challenges shaping the children’s pathways during this period and to consider attachment as part of this overall development. Universally, children’s responsibilities change, while their social ties transform. However, each of these developmental tasks has context-specific translations, depending on the specific features of the developmental environment (Weisner, 1984). Firstly, children during middle childhood are faced with increased responsibilities and agency while being introduced into new settings (Weisner, 1984; LeVine and New, 2008). For children in rural traditional areas, this translates into their new role as caregivers in the system of shared care, becoming responsible for others (Weisner, 1984). Traditionally, they are also introduced into adult settings of their environment, participating in the workforce, but also in religious settings, e.g., secret societies (Ndze, 2008). For children in Western contexts, this increased responsibility concerns an expectation of self-regulation and independence, mostly in the new setting of school, with children becoming more self-reliant. They spend more time with peers, while their parents continue to supervise and manage their behavior (Weisner, 1984). Secondly, their social setting changes with children now having to develop and improve their skills to establish and maintain relationships, especially those with peers (Weisner, 1984; Kerns and Brumariu, 2016). While children in traditional rural areas generally do not have to construct new relationships, having been integrated into a tightly knit interconnected community from early on, they must now independently regulate and sustain their ties to peers as parental supervision rapidly decreases. This normative parental withdrawal, beginning from the point of weaning, is compensated by an inclusion into peer groups, further increasing the importance of peers in these contexts (Weisner and Gallimore, 1977; Weisner, 1984; Gottlieb, 2004; Lancy, 2015). Children in Western contexts are increasingly confronted with unknown peers and adults and have to establish new friendships and formal relationships, also increasingly less with the support of their parents (Weisner, 1984; Harkness and Super, 1992).

Based on this current state of research on children’s psychosocial development during middle childhood, this study aims to extend the investigation into the adaptiveness of attachment beyond the first years, focusing on children’s attachment figures. Combining the identification of these individuals with a functional perspective, we explore *who* provides children with a feeling of security and *how* this feeling is established during middle childhood. In order to consider both contextual adaptiveness and universal patterns, we compare data from two settings, representing a traditional and a Western environment of childhood and include each developmental context in its distinctive setup. By making the contexts an inherent part of the study, we are able to explore and discuss links between empirical data on children’s attachment patterns and the distinctive contextual characteristics of each setting. Introducing a network perspective on attachment during middle childhood, we aim to identify all attachment figures, overcoming the limiting hierarchical focus on superordinate parental attachment figures, resulting from monotropic concepts of attachment. The exhaustive assessment allows us to explore the complexity of the entire resource of attachment, investigating the size and the composition of this collective of individuals establishing a feeling of security. By also adopting a functional perspective, we are able to understand if children distinguish between various domains of security and if they allocate these responsibilities to different attachment figures, with members of children’s attachment networks possibly cooperating to jointly generate a comprehensive feeling of security.

Methodologically, we aim to implement a child-centered approach with the children’s own concepts of attachment and security guiding our research while combining psychological research and ethnographic methodology. We endeavor to generally reduce any possibly restricting preselection or specifications since we do not share the children’s age group or even cultural background.

Due to varying cultural rules of emotional display (Keller, 2014), we focus on the attachment figures’ role of a secure base in this study, thus defining attachment figures as individuals who provide a continuous feeling of security and comfort, enabling free exploration (Ainsworth, 1989).

To investigate the selection process, children do not only identify these individuals, but also characterize them on socio-structural dimensions, focusing on age, gender and physical proximity, selected for their importance in structuring interactional patterns in both contexts and their previously demonstrated influence on the children’s selection of general social relationships (Epstein, 1989; Ruble et al., 2006). Children also characterize their individual ties concerning relationship stability, with long-term continuity previously only been ascribed to parental ties, to empirically investigate the influence of this feature on the children’s selection and to assess its distribution across all ages of attachment figures. Additionally, children are asked to characterize these attachment figures concerning their perceived functionality in order to assess the respective roles of individual attachment figures in maintaining a feeling of security and to further investigate children’s understanding of security and their perception of context-specific sources of (in)security.

## Materials and Methods

### Samples and Recruitment

We selected two settings, each of which constitutes an example for the prototypical sociodemographic settings used to compare and contrast childhood development: Nseh in Cameroon as an example of a traditional, relational village, and Bad Nauheim in Germany as an example of a post-industrial Western middle-class setting.

The 11 participating children of Nseh (6 girls, 5 boys) between the ages of 6 and 10 years were recruited with the help of our local research assistant. After receiving a written consent by the clan’s leader, adult guardians and children individually gave written consent in accordance with the Declaration of Helsinki to voluntarily participate after receiving information on the study. Introductory sessions and interviews in Pidgin English and Lamnso, the local language, took place in the children’s free time.

The 11 children of Bad Nauheim (six girls, five boys) were recruited at a primary school, selected to match the sample of the Nseh in gender and age range. The sample only includes children without a migration background, so as to assess data from a homogenous sample of families with a shared cultural model. Again, both children and parents then gave written consent to voluntarily participate in the study. Introductory sessions and interviews took place during school hours.

The study was carried out in accordance with the recommendations of the ethical guidelines of the German Association of Psychology. The protocol was approved by the ethics committee of the Department of Psychology of the Goethe-University Frankfurt.

### Comparative Description of the Settings

While cross-cultural comparisons of extreme settings highlight contrasting differences in developmental patterns, they generate oversimplified explanations for observed patterns when only considering the impact of abstract cultural dimensions, neglecting the complexity of childhood environments (LeVine and Norman, 2008). Thus, the two selected contexts are understood as examples for extreme sociodemographic settings, while we also take their individual setup into consideration, aiming to take a closer look at both contexts of childhood on a variety of dimensions, implementing the outlined ‘ecologically informed’ path to attachment research. Following this approach, we are able to jointly highlight the range of developmental pathways of attachment during middle childhood, while also reflecting on the context-specific processes of adaptation.

The selection of contextual dimensions is guided by Keller’s ‘ecocultural model of child development,’ focusing on the influence of physical environmental structures, population parameters and socioeconomic structures on child development (Keller, 2007, p. 31; also Keller, 2014). This model is based on J. Whiting’s research on contextual attributes shaping children’s learning environments across cultures, differentiating between the environment, the history and the maintenance system of a setting (Whiting, 1981). Concerning the physical environment, we were also guided by the ideas of Whiting and Edwards (1988) on the importance of the objective setting of development, focusing on the distinctive space and composition of children’s real-life developmental environments. We decided to additionally investigate the children’s daily schedule in order to focus on their interactional patterns throughout the day (Whiting and Edwards, 1988; Weiss, 1993; LeVine and Norman, 2008).

While these dimensions only constitute an excerpt of the multidimensional contexts, we are able to discuss if patterns in children’s attachment networks correspondent to these contextual factors, aiming to develop an exploratory explanation.

#### Physical Environment and Population Parameters

Nseh is a village and a clan in the northwest region of Cameroon. It neighbors several larger clans like the Nso, a setting that has already been thoroughly investigated concerning children’s development in the early years (Nsamenang and Lamb, 1993, 1995; Otto, 2008). These powerful neighbors are still considered a subtle threat to the autonomy and existence of the Nseh. While having adapted to the languages of their neighbors after several migration movements and having become an administrative part of the Nso through colonial demarcation, the clan of the Nseh identifies as an independent social, cultural and political entity (Fjellman and Goheen, 1984; Ndze, 2008). The village is situated in the volcanic highland of Cameroon and surrounded by farm land and eucalyptus trees which are used as firewood. The historical narrative of the clan ascribes its origin to a common ancestor whose successor is the respective ruler or *fon* of the Nseh, holding political, religious and financial power (Ndze, 2008).While there have not been any official censuses, there are about 20,000 members of the clan living in Nseh, according to estimates. Despite this size, Nseh is considered to be a village both administratively and in the self-concept of its residents. The village covers a large stretch of land and is divided into several quarters according to the lineages of the clan. Each of them have their own traditional administration, subordinate to the main ruler. Most commonly, several families with close kin ties share the same residence. This dwelling unit is defined by distinctive borders, with roads or pathways marking the limits of the compound. Each nuclear family, i.e., parents and the biological and social children, possesses an individual sleeping house on the compound, while kitchens, parlors and open court yards are shared by all members of the compound family. These are also the areas where most activities during the day take place, resulting in a close interconnectedness and interdependence of the compound family. Thus, children consider all other children from their compound to be their siblings. Despite the increase in population resulting in urban densification in the general region, Nseh remains a spacious village. Thus, sleeping houses are mostly scattered on large compounds with lots of free space in between. In our sample, there are, on average, 16 individuals (ranging from 6 to 23 members per residence) coming from 3.1 elementary families who share the same residence. The mothers of the sample have an average of 5 biological children (ranging from 1 to 10 children) who are sometimes complemented by social children. They were 20.2 years old when they had their first child (ranging from 15 to 30 years of age). Seven out of 11 fathers live with their children or support the mother.

Bad Nauheim is a town situated in the lowlands of central Germany, approximately 30 km north of the city of Frankfurt/Main and thus an extension of the Rhine-Main region. The outskirts of the town are shaped by agricultural areas, remains of the former agricultural focus, which are progressively converted into housing estates. Once a Celtic settlement that profited from the rich soil of the area throughout centuries, the modern history of the town has been shaped by its medical spring, which expanded into a luxurious Art Nouveau spa in the 19th century. With about 30,000 inhabitants, the town is divided into a central part and five suburbs, each of them having their own administrative structures subordinate to an elected city council (Stobbe, 1997; Rippel, 2011). The sample of Bad Nauheim was assessed in one of the more affluent suburbs. Most nuclear families live in one-family residences built on large plots. Since the near metropolitan area generally results in a densification of residential units, these plots become increasingly smaller for families with newly built houses. Again, individual residences are clearly marked by roads, fences and pathways. Thus, children make a clear-cut distinction between their biological siblings and all other children. The nuclear families of the sample consist of an average of 4.2 family members (ranging from 3 to 6 members). Mothers of the sample have an average of 2.18 children (ranging from 1 to 4 children). They were 32.3 years of age (ranging from 27 to 42 years) when they had their first child. All children reported to live with both their biological parents. The majority of families have their grandparents living in the same suburb, a few even in the same residence.

#### Socioeconomic Parameters

##### Economic situation

The general infrastructure of Nseh is rather poorly constructed, with most of the homes in the village not connected to electricity or a water supply system. It is further impaired by the rough climate with rains and dry periods each lasting for months dissolving roads either into mud or dust. While the health infrastructure has improved, leading to a decrease in infant and maternal mortality, the morbidity rate among children remains high due to the costs of medical care, with children commonly suffering from malaria and respiratory diseases. The economy of Nseh is based on subsistence farming. Situated in a thick forest with a large variety of wildlife only decades ago, all available land has been transformed into agricultural areas with the quality of the soil slowly deteriorating due to a constant cultivation. The general agricultural situation is characterized by a high degree of insecurity since there is only a single yearly harvest so that surprising losses due to the weather or other unforeseen circumstances have a major impact. As a result, food supply is considered to be critical, making rationing necessary at the end of the crop year with children during middle childhood well aware of this situation. Foreign investors constitute an additional threat with land grabbing and the discovery of noble earths in the region. The former cash crop of coffee is no longer profitable due to the low world market price. Almost all families have an additional income, with parents working as teachers, drivers or craftspeople. Most mothers only have primary education (64% of the investigated mothers), few have additional years of education (18% of the investigated mothers) or even a degree of secondary education (18% of the investigated mothers). Children during middle childhood constitute an active part of the work force, especially at the farms, while having a multitude of chores in the compound.

Being part of a metropolitan area, the general infrastructure of Bad Nauheim is reliable and comprehensive, including low-threshold access to a highly specialized medical infrastructure in the region. All children in the sample from Bad Nauheim sample live in families of academics with parents working either full or part-time jobs, if not on maternity leave. The nearby city of Frankfurt offers a wide range of highly paid jobs in financing and engineering. The macroeconomic situation of the area and in overall Germany is thriving with a very low rate of unemployment among university graduates (Bundesagentur für Arbeit [Federal Employment Agency], 2017). However, this situation also leads to an increase in the costs of living (Hessisches Statistisches Landesamt [Statistical Office of Hesse], 2017). The German welfare system provides additional stability in case of any unforeseen circumstances (Bundeszentrale für Politische Bildung [Federal Agency for Civic Education], 2015). Only few children are responsible for any chores in their parental household, most of them supporting their parents irregularly and on their own accord.

##### Social structures

The social structure of the clan of Nseh is hierarchically organized and based on age and kin, with most children during middle childhood being able to explain their individual kin ties in detail. The overall clan is divided into several lineages, that traditionally live in separate quarters as mentioned above. These quarters are further separated into compounds. For the Nseh, closeness of kin commonly translates into residential proximity with the closest in kin living in the same compound and neighboring compounds sharing decreasing kin ties (Ndze, 2008). Closeness of kin is generally known to translate into trust and sociability, but also into social obligations (Hahn, 2012), resulting in reciprocal support provided by those with close kin ties. Children during middle childhood help their extended families on their farms or offer assistance as a babysitter while receiving moral, financial and provisional assistance especially during times of need. With interactional patterns of the Nseh also based on a system of seniority, age differences translate into strict behavioral norms of power and respect, comprehensively subordinating children to adults even in everyday life (also cf. Nsamenang and Lamb, 1993 for the neighboring Nso). Gender does not only influence the distribution of formal power with each level of the kin-based social hierarchy headed by a male clan member, responsible for all life-altering decisions in his branch of the family. It also shapes the distribution of labor in the compound and at the farm. Men take over strenuous, but infrequent tasks, while also having sacral responsibilities. Women are responsible for everyday routines, especially concerning child care. Women’s groups that save and loan money to their members, however, provide additional financial independency and support structures of social power that subvert the patriarchal order of the clan (Becke and Bongard, 2018b).

In the German sample of Bad Nauheim, residential structures are likewise built on kin with elementary families on their individual plots constituting rather independent social units that are, however, closely connected to grandparents and other close relatives. While there is a slight connection between kinship and residency with grandparents of some families living in the same suburb, this is not a regular pattern in the setting. Besides these kin-based ties, members of the community are linked through friendship and shared social or professional activities. The overall community exhibits hierarchical levels built on income and social class. Being an upper middle-class suburb, the families of the sample have rather homogenous class affiliations. While age differences structure interactional patterns in families and allocate responsibilities, resulting behavioral norms are commonly not enforced in a strict way, with children contributing to decision in everyday life. Despite both parents of most families having comparable academic degrees, a continuous gender-based income disparity lowers women’s economic power in the family. Social power in the families is equally distributed, but most household chores are still assigned to the mothers.

##### Daily schedule

In the morning, children of Nseh have breakfast in the shared kitchen area before finishing their chores in the compound. After that, they attend compulsory primary school until noon. However, not all parents prioritize their children’s formal education, as they are disappointed in a corrupt school system and low academic standards. Parents mostly leave to their farms or other work places in the morning only to return in the late afternoon. After school, children mostly move around in rather large peer groups, with little to no immediate supervision or contact to adults; this situation results from age-based hierarchical boundaries and interactional restrictions in the clan. For the neighboring Nso, peer groups have been described as an important setting of children’s socialization beginning from the point of weaning. Despite their free climate, they adhere to societal norms set by adult clan members (Nsamenang and Lamb, 1993; Nsamenang, 1994). For the Nseh, this translates into a limitation of movement of these peer groups to their immediate environment within their kinship ties, following interactional norms set by adult clan members that rely on kinship as a marker of trustworthiness. This is enforced by the spacious nature of the village and the lacking access to any means of transportation, mostly keeping children from breaking these bounds Thus, children during middle childhood only cover larger distances when sent on errands by adults. During the afternoon, peers join hands to fulfill their assignments in the compound, but also play games. These peer groups include children of middle and early childhood, since children of middle childhood are now oftentimes responsible to care for their younger siblings. Peer groups also constitute the only legitimate context of emotional expressiveness for children during middle childhood due to the parental withdrawal after the first years (Becke and Bongard, 2018b). In the evening, the family gathers in the kitchen to prepare and consume a shared meal and receive visitors.

In Bad Nauheim, children leave to primary school after having breakfast in their elementary families, with most children reporting that both parents are present in the morning. Since grades at the end of primary school dictate the following educational track, having a long-term impact on career opportunities, primary school education is very important to most parents. In the afternoon, most children participate in additional pedagogical and institutionalized structures of education, attending after-school care, music lessons or sport clubs. With grandparents close by, children also regularly spend time with them, when parents are working long hours. In their free time, they meet in pairs, or rather small groups of friends, with such meetings often planned ahead, sometimes even arranged by their parents. Having access to bikes and scooters, children are able to cover larger distances within the entire suburb. They also regularly spend their spare time playing or taking excursions with their parents, with relationships between adults and children less influenced by age-based hierarchical concepts. Most children report that their mothers are at home during the afternoon, while fathers only return in the evening for a shared family meal or even after the child has gone to bed.

### Measures

To firstly assess sociodemographic information on children and their environment, we conducted individual guided interviews with all children of both samples, asking them to describe their family structures, including the kinship ties, the individuals’ ages and occupations, and to detail their daily routines in their families and beyond, considering activities in institutional settings and in their free time, as well as their chores.

Secondly, we conducted photo elicitation interviews (PEIs) to assess and differentiate children’s attachment figures (Becke and Bongard, 2018a). PEIs allow children to express their own perception, bridging the cultural and age-based gaps of understanding (Barker and Weller, 2003; Samuels, 2004; Montgomery, 2009; Luttrell, 2010). The cross-cultural applicability of PEI has been demonstrated numerously, attributable to the open character of the measure (e.g., Clark-Ibáñez, 2004; Samuels, 2004; Gotschi et al., 2009; Luttrell, 2010). We also were able to select PEI since children of both contexts were well acquainted with the concept and the process of taking pictures, with cameras being objects of everyday life in both settings. While children of the Nseh had less individual practice than children in Bad Nauheim, they were more familiar with analog cameras.

During introductory sessions, children received a disposable camera and the identification task, together with rules to ensure privacy during the assessment process. We asked:
Standard English: “*Please take pictures of anyone that is important to you, anyone with whom you feel safe, comfortable and at ease.”*
Pidgin English: *“Abeg, take photo with any man way e be important for you, any man way you feel safe, comfortable and free with e.”*
Lamnso: *“Kiì wò ku lì vindzЭЭdzЭm ve wìr vi, a fЭm dzЭ vЭné wo wù dzЭЭn e shií fo wò, wìr wo vèn wun lòo dzЭ a wàa dzЭ là fan, a dzЭ kijuη e kfЭn a dzЭ kicaarsin.”*
German: *“Bitte fotografiere alle Personen, die dir wichtig sind, bei denen du dich sicher, wohl und unbeschwert fühlst.”*

These translations were each developed in cooperation with bilingual experts. All children received a detailed explanation on how to use the analog camera and took a first picture during the introductory session. We highlighted that they were free to take as many pictures as they needed without feeling obliged to use all of the film. During the second step, follow-up interviews were conducted guided by the pictures children had used to identify their attachment figures, with children providing socio-structural allocations of their attachment figures concerning age, gender, physical proximity and the duration of the tie and characterizing the individual functionality of each tie.

While most previous studies only differentiated broadly between ‘peers’ and ‘adults’ concerning the age of attachment figures, children were asked to differentiate peers according to their developmental stages of early and middle childhood and adolescence. This was done so using the milestones of entering primary and secondary school as a cut-off line in the German setting, while following the indigenous model of progressive developmental stages in the Cameroonian context, with these stages mostly harmonizing with Western concepts of childhood stages^1^ (Becke and Bongard, 2018b).

Children also categorized their attachment figures according to the closeness of their residence for us to understand if physical proximity and the resulting convenience of accessibility influences the inclusion of an individual into children’s networks. We differentiated between units that allow children to independently approach these attachment figures, i.e., sharing the same main residence, sharing the immediate neighborhood, conceptualized as a walk of less than 5 min, or sharing the same town, and those units in which people can only be visited by parental mediation, i.e., living in a different town or even abroad. While the composition of dwelling units differs between both settings, they are marked by distinctive borders and a clear decrease of interconnectedness beyond these boundaries in both settings. Thus, it can be assumed that children have a similar concept of a “shared residence.” Since the concept of a “shared neighborhood” potentially differs vastly between both contexts, not having a comparable landmark, we based this category on physical distance, specifically the time needed to cover it.

Additionally, children characterized the stability of each attachment relationship, stating their own developmental stage when constructing the tie, thus categorizing whether or not they had known the individual from birth, before or after the age of 6 or if they had only met the individual within the last year^2^.

To also assess perceived functionality, we asked:
Standard English: *“Why did you include this person? How does he/she make you feel safe and comfortable?”*
Pidgin English: *“Why you na take this person? How e dey make you feel safe and comfortable for e corner?”*
Lamnso: *“Bì‘ka mo a ki sho’on wír vЭn? Wù lo wù ghàn é le bóo yiì aà wáa dzЭ là fan, e kfЭn a dzЭ kijuη”*
German: *“Warum hast du diese Person ausgewählt? Wie gibt sie dir ein Gefühl von Sicherheit und Wohlbefinden?”*

### Data Handling and Analysis

Attachment figures were identified following the children’s statements on whom they had purposefully photographed. We included pictures of representative objects if children indicated this in their interviews. Some children also added people as part of their attachment networks during the interviews. These few individuals were also included into the analysis. As our main focus is on the children’s own perception, their verbal statements are considered to be of the same relevance as the pictures taken.

Children’s responses to the perceived functionality of each attachment tie were coded separately for both samples using ethnographic strategies to detect data-immanent patterns (Spradley, 1980/2016) combined with a shortened approach to grounded theory (Corbin and Strauss, 2015), developing a set of categories to understand how individual attachment relationships provide security in both settings.

Across both samples, 278 attachment figures were identified and structurally and functionally characterized. Frequency distributions of these 278 attachment figures then built the foundation for further data analysis using a t-test to compare the network size across settings, as well as χ^2^-tests and Fisher’s exact tests for the socio-structural and functional dimensions of children’s networks.

## Results

### Identification and Socio-Structural Characterization of Children’s Attachment Networks

A two-sample *t*-test indicates that children of the Nseh (*M* = 15.9, *SD* = 2.68) report significantly larger attachment networks than children in Bad Nauheim (*M* = 10.6, *SD* = 2.29), *t*(277) = 17.66, *p* < 0.001, *d* = 2.13).

**FIGURE 1:**
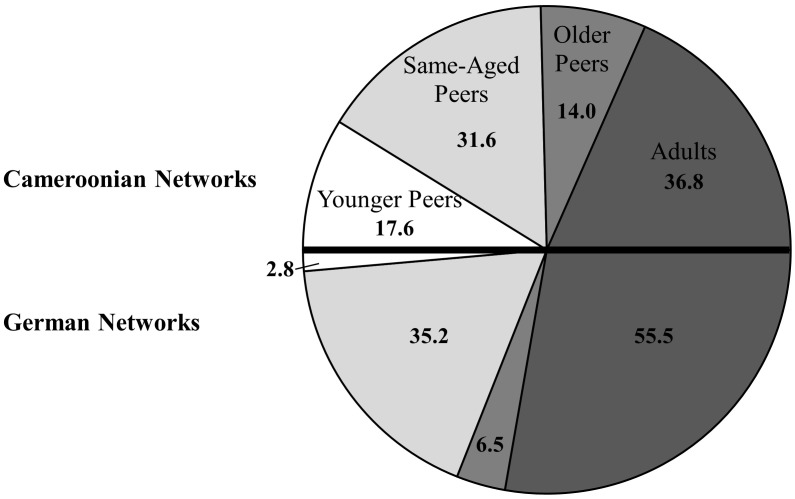
Distribution of age categories in both samples.

Age influences the inclusion of individuals into children’s attachment networks in both settings [for the Cameroonian sample of the Nseh χ^2^(3, 171) = 24.58, *p*< 0.001; for the German sample of Bad Nauheim χ^2^(3, 108) = 80.96, *p*< 0.001]. Both settings significantly differ in their distribution of age [χ^2^(3, 279) = 21.12, *p*< 0.001]. Peers during middle childhood, i.e., same-aged, are important for both samples, while adults play a larger role for children in Bad Nauheim. Children of the Nseh report to also rely on younger and older peers, while those are almost irrelevant for children in Bad Nauheim (see **Figure 1**^3^ and Supplementary Table 1).

Concerning gender, it neither influences the inclusion of an individual into children’s networks in either setting [for the sample of the Nseh χ^2^(1, 171) = 1.69, *p* = 0.19; for the sample of Bad Nauheim χ^2^(1, 108) = 2.37, *p* = 0.12], nor do both settings differ in their composition concerning gender [χ^2^(1, 279) = 0.16, *p* = 0.69]. However, the boys and girls of both samples show a significant preference for same-aged attachment figures of their own gender [χ^2^(1, 48) = 12.00, *p*< 0.001 for girls; χ^2^(1, 44) = 9.09 *p* = 0.003 for boys). This preference does not apply for any other age group, as all other comparisons did not reach statistical significance with all χ^2^< 3.38 and all p > 0.09 (also see Supplementary Table 2).

**FIGURE 2:**
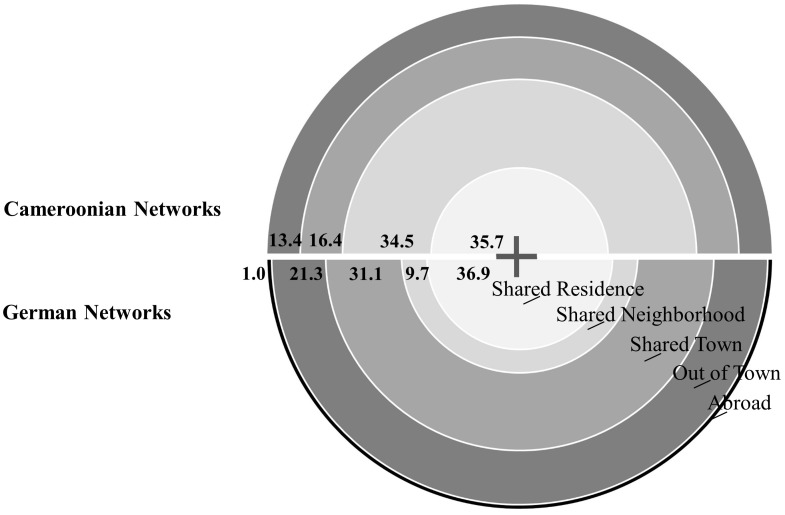
Distribution of residential categories in both samples.

The physical proximity or closeness of residence influences the inclusion in both settings [for the sample of the Nseh χ^2^(3, 171) = 28.18, *p*< 0.001; for the sample of Bad Nauheim χ^2^(4, 103) = 45.20, *p*< 0.001]^4^, while there are also significant differences between the samples [χ^2^(4, 274) = 26.16, *p* < 0.001]. Children in the sample of the Nseh report rather narrow networks, focusing on the closest categories with relevance strongly decreasing as distance increases. Children in the sample of Bad Nauheim report rather broad networks, with many attachment figures coming from close physical proximity, however, complemented by individuals from more distant residential structures, only sharing the same town or even living out of town (see **Figure 2** and Supplementary Table 3).

**FIGURE 3:**
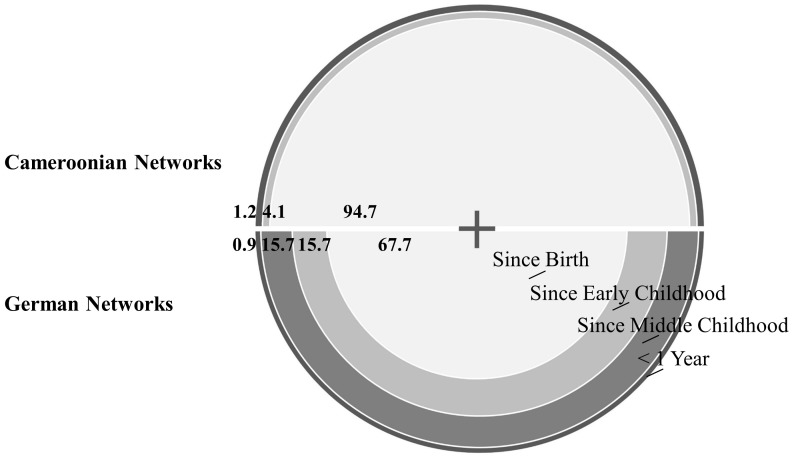
Duration of individual attachment ties in both samples.

The duration of a tie significantly influences the individual’s inclusion in both settings [for the sample of the Nseh χ^2^(2, 171) = 290.35, *p*< 0.001; for the sample of Bad Nauheim χ^2^(3, 108) = 110.82, *p*< 0.001], highlighting the importance of relationship stability for the children’s selection. Both settings also differ in their patterns (*p* < 0.001, Fisher’s exact test). For both samples, most relationship ties originate from the children’s earliest developmental stage, while only few attachment ties have evolved in the last year, highlighting the importance of stability for attachment. Children in Bad Nauheim, however, describe an expansion of their social networks over their development with a considerable number of ties only originating from early or even middle childhood (see **Figure 3** and Supplementary Table 4).

Concerning the distribution of stability across the age categories of the attachment figures, almost all relationships exhibit long-term stability in the sample of the Nseh. Thus, there is no significant relation between the individual attachment figure’s age and the duration of the tie (*p* = 0.61, Fisher’s exact test).

For the sample in Bad Nauheim, there is a connection between duration and age categories (*p*< 0.001, Fisher’s exact test). Almost all adult ties are marked by long-term stability, as well as those to older peers. Ties to same-aged peers mostly result from the different stages of childhood, having a varying degree of restricted stability. However, there is a considerable number of ties to same-aged peers that stem from the earliest developmental stage (see **Table 1**).

**Table 1:** Classification into duration categories for each age group in both settings as percentages.

Duration category	Cameroonian sample of the Nseh					German sample of Bad Nauheim				
	Younger peers *n* = 30	Same-aged peers *n* = 54	Older peers *n* = 24	Adults *n* = 63	Total *N* = 171	Younger peers *n* = 3	Same-aged peers *n* = 38	Older peers *n* = 7	Adults *n* = 60	Total *N* = 108
From birth	93.3	92.6	100	95.2	94.7	66.7	26.3	71.4	93.3	67.6
From early childhood	6.7	3.7	–	4.8	4.1	–	44.7	–	–	15.7
From middle childhood	–	–	–	–	–	–	29.0	28.6	6.7	15.7
Less than 1 year	–	3.7	–	–	1.2	33.3	–	–	–	0.9

*Percentages relate to the individual age groups.*

### Functional Characterization of Children’s Attachment Networks

#### German Sample of Bad Nauheim

For the sample of Bad Nauheim, five categories of functionality emerged, with each child employing at least three different categories to characterize their attachment figures while commonly attributing a single domain of security to each attachment figure.

##### Socio-emotional support

In the most common category of perceived functionality, children reference the ability of their attachment figures to help them to emotionally cope with socially difficult situations, thus providing a resource that the child does not (yet) have. The individual mediates the solution of the problem and re-establishes emotional balance, offering comfort or encouragement. *“Because he is nice and he can comfort me. When someone isn’t nice, he does not become agitated but rather asks them to stop with their fight and to calm down,”* -a boy talking about his father).

##### Consistency

Relationships in the second most common category provide a feeling of comfort and security by means of their comprehensive character, compassing several areas of life or several developmental stages, thus providing a consistent baseline or a ‘secure base’ *(“I feel safe 24/7. I have known him from my birth, he is a part of my life,”* -a girl talking about her father).

##### Harmonization

These descriptions in the third most common category reference the reciprocal ability to find a harmonious agreement and to attune to each other in a conflict-free, trusting and homogenous relationship, resulting in agreement (*“We can always settle on what to play and he always agrees to play whatever the other person wants to play. He knows how to get along well with anyone,”* -a boy talking about his same-aged friend).

##### Education

In such relationships of the fourth most common category, children reference the individual’s support in their education as a source of comfort and security. They describe attachment figures as offering relief concerning school work, outlined as a stressor *(“When I entered primary school, he was already in class 4. He helped me with my homework and also with finding my class rooms. I will now transfer to a secondary school where he already attends class 8. Again, he will help me there,”* -a girl talking about her older brother) or as imparting knowledge outside of formal contexts, this education being described more favorable.

##### Instrumental assistance in distress

Individuals assorted to the least common category of perceived functionality elicit a feeling of comfort and security by providing immediate instrumental help in physically challenging situations, ranging from accidents and illnesses, to injuries. “*We were on a boat ride on a river and I fell from the banana toy and hurt my arm and my knee and I fell into the water and he pulled me back unto the boat,”* -a girl talking about her father).

**FIGURE 4:**
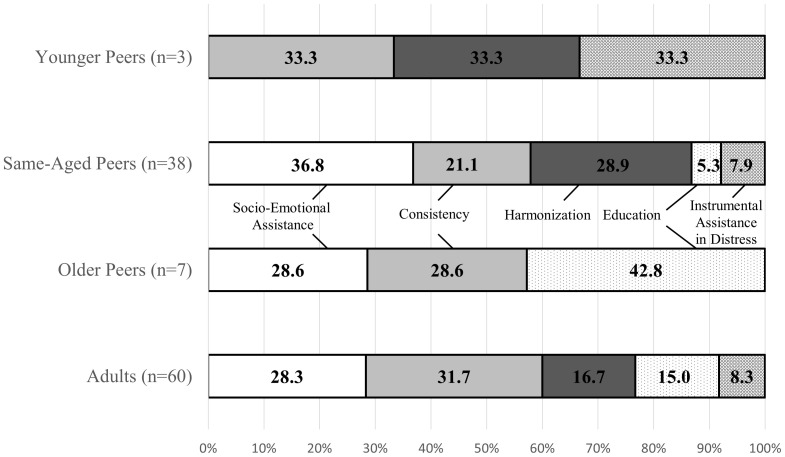
Categories of perceived functionality across age categories in the German sample of Bad Nauheim

For the sample of Bad Nauheim, the distribution of perceived functionality is neither influenced by gender [χ^2^(4, 108) = 1.49, *p* = 0.83], nor by age (*p* = 0.18, Fisher’s exact test). The categories of socio-emotional assistance and consistency are relevant across almost all ages, as well physical protection in distress; this category, however, mostly to a lesser extent. Children value harmonization mostly in their relationships to same-aged and younger peers; these, however, are only represented by a rather small subsample. Education is mostly referenced when talking about attachment ties to older peers and, to a lesser extent, to adults, while almost irrelevant concerning same-aged peers (see **Figure 4** and Supplementary Table 5).

#### Cameroonian Sample of the Nseh

For the sample of the Nseh, four categories of perceived functionality emerged when coding children’s responses, with children using between three and all four of them to describe the functionality of their attachment ties. Again, children only attributed a single domain of security to each attachment figure.

##### Nutritional care

This most common category describes all statements that explicitly reference a supply with food. These attachment figures offer a resource the children cannot independently access. Children either reference a unidirectional provision for older peers and adults, or the process of sharing food for same-aged and younger peers (“*He catches birds and prepares them and when I am passing, he will give me a share*,” -a girl talking about an older peer from her family).

##### Kinship

In these statements of the second most common category, children describe a kin-based tie to their attachment figures referencing broad categories of kinship or describing in great detail the exact relationships (“*They are my younger ones*,” -a boy describing the relationship to his younger siblings).

##### Everyday instrumental assistance

In these statements of the third most common category, children describe their attachment figures as providing instrumental assistance in situations of everyday life, especially concerning their chores in the compound and at the farm, sometimes explaining the reciprocity of assistance, but never relating to situations of distress (“*When I send her to go and carry water, she will go and bring the water and I will wash her dress*,” -a girl talking about her younger sister).

##### Affection

In these statements of the least common category, children talk about friendship and the explicit emotional value of their attachment ties in broad terms (“*They are my friends*,” “*I like them*,” -a boy talking about his friends with a kinship tie), sometimes referencing their own emotional value for younger peers.

**FIGURE 5:**
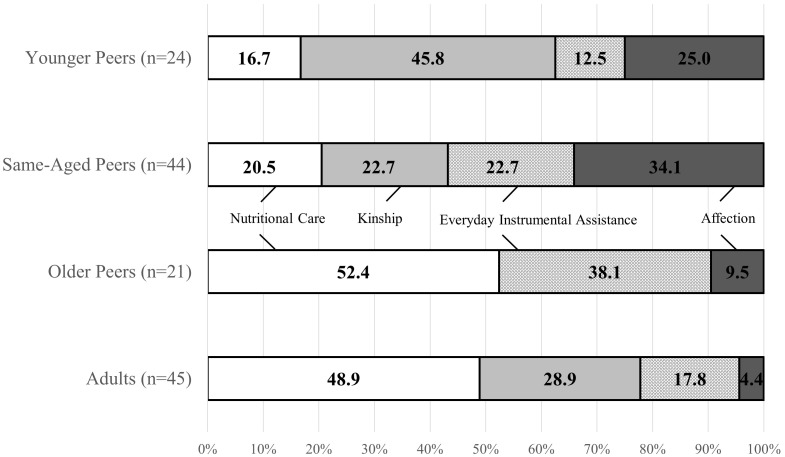
Categories of perceived functionality across age categories in the Cameroonian sample of the Nseh.

Again, the gender of the individual attachment figure does not influence their perceived functionality [χ^2^(3, 134) = 5.51, *p* = 0.14]^5^. For the sample of the Nseh, however, the individual’s age influences their perceived functionality (*p* < .001, Fisher’s exact test). Nutritional Care is mostly referenced when talking about older peers and adults. Kinship is the most important category for younger peers, but also of great importance for adults. Instrumental assistance is a relevant category when talking about older peers. Affection is most important concerning same-aged peers and also of great relevance concerning younger peers, while children scarcely reference this category when describing attachment relationships to older peers and adults in the follow-up interviews (see **Figure 5** and Supplementary Table 6).

## Discussion

Overall, children across contexts display considerable differences *whom* they identify as their attachment figures and *how* these ties provide a feeling of security. Since we included an in-depth investigation of both developmental settings in our study, we can now reflect on the contextual adaptiveness of these patterns, exploring links between empirical data and contextual features in order to understand how the observed attachment networks are shaped by ecocultural conditions.

### The Adaptiveness of the Network Size and the Socio-Structural Patterns Across Contexts

Children of both contexts possess a diverse attachment environment during middle childhood, presumably resulting either from a system of shared care in the Cameroonian setting of the Nseh or the social expansion during middle childhood in the German setting of Bad Nauheim. Thus, children across both samples nominated a variety of individuals as their attachment figures. This data demonstrates the complexity of the resource of attachment and the importance of a network approach with monotropic research strategies unable to adequately investigate the collective in its entirety. Children of the Nseh, however, described comparably larger networks, possibly resulting from both the continuously larger social environment they experience from infancy on and their socialization toward interconnectedness.

Taking a closer look at the socio-structural allocation of the identified attachment figures to understand the selection process, the distribution of age categories mostly reflects the distinctive care system of each setting. Since child care is mostly an adult and especially parental task in the Western care settings, children in Bad Nauheim report to predominantly rely on adults. Being raised in a more diverse care system that includes peer care, children of the Nseh also nominate older peers. Furthermore, they frequently nominate younger peers. Since it has been pointed out for other African contexts that taking care of younger siblings can contribute to the children’s value of social responsibility and to their sense of agency (Serpell et al., 2011), future research will need to consider how sibling care and the children’s position of catering to the need of attachment of their younger peers could also contribute to their own feelings of security, possibly resulting in comfort in both partners of these attachment ties. However, children across both samples also identify a considerable number of same-aged peers as their attachment figures, i.e., individuals contributing to their continuous feeling of security. For the context of the Nseh, these peers constitute an important developmental and interactional space, resulting from the normative parental withdrawal during this period, as demonstrated for many comparable Sub-Saharan settings (Weisner and Gallimore, 1977; Weisner, 1984; Gottlieb, 2004; Lancy, 2015). For the context of Bad Nauheim, however, this pattern contradicts previous reflections of adult researchers on attachment during middle childhood that mostly exclude peers from closer investigations based on the preset concepts of relevance of attachment ties, while not giving children the possibility to explain their own perceptions of security. Yet, the pattern also surprisingly strongly contradicts results of the only study that thus far allowed children to identify the entirety of their attachment figures, generally only referencing a small number of mostly adult attachment figures (Seibert and Kerns, 2009). We assume that these differences concerning size and composition possibly emerge due to our broad identification task, guided by the feeling of security, with children nominating anyone who establishes and maintains this feeling compared to the situation-based task used by Seibert and Kerns (2009), with children nominating individuals to whom they would turn in a specific pre-selected situation (being away at a summer camp, having a fight with a friend) or in the event of a pre-selected negative affect (sadness, anxiety). This situation-based task explicitly follows Bowlby’s reflections on attachment behavior during infancy, which predominantly focuses on physical proximity as the function of attachment behavior (Bowlby, 1969). The task could possibly be understood as a (functional) restriction by participating children, with this approach thus only depicting an excerpt of children’s attachment networks, deviating from the more current broad self-concept of attachment theory, which focuses on the general “need for security” (Hazan and Shaver, 1994, p. 9). That concept considers “felt security” as the main function of attachment behavior (Sroufe and Waters, 1977, p. 3, also Ainsworth et al., 1978; Hazan and Shaver, 1994; Mikulincer et al., 2003) and thus takes the growing complexity of attachment behavior into consideration, which includes distant and symbolic interactive patterns from middle childhood on (Bowlby, 1969; Ainsworth, 1989; Raikes and Thompson, 2005; Kerns and Brumariu, 2016). Being independent of distinct behavioral patterns that vary across cultures, that approach, focusing on the feeling for security, also facilitates a cross-culturally valid assessment of attachment. Additionally, it is difficult to compare patterns across both studies because little to no information on the particular developmental setting is provided by Seibert and Kerns (2009) in order to contextualize their data and identify characteristics in the setting which contribute to the different patterns. Thus, we cannot easily assume that both studies assess data in a comparable ecocultural context. Our context could possibly differ from their research setting concerning the possibilities of interactions with peers and the consistency of these relationships over the children’s development, resulting from educational structures and care settings after school and even the degree of social interconnectedness of neighborhoods, potentially varying between small, close-knit towns and anonymous urban settings.

Concerning gender, both settings demonstrate a different pattern in the distribution of care responsibilities, power and labor. While women in the setting of the Nseh are responsible for the majority of child care and household chores, they hold only little to no formal power. In the setting of Bad Nauheim, children experience equal contributions to care, at least concerning the quality and the effort of care, while not always concerning the amount of time, and an almost equal distribution of formal power between parents. Surprisingly, these differences between the two contexts do not influence the children’s selection of their attachment figures across genders. The only gender preference occurs concerning same-aged peers in both contexts, in line with previous research on peer selection (Ruble et al., 2006). Future research in the setting of the Nseh will need to determine why especially the different contributions to child care across genders do not influence children’s selection of adult attachment figures.

The influence of physical proximity and accessibility seems to depend on the legitimate interactional space of each setting. The Nseh generally rely on kinship as a social concept of interconnectedness. In the residential structure of the clan, closeness of kin translates into physical proximity, making the immediate environment, i.e., the compound and the neighborhood, the legitimate interactional space in which children can freely move from early on, resulting in narrow networks and a sharp decrease in relevance beyond this environment. These limitations are increased by the lack of personal transportation. In the setting of Bad Nauheim, the legitimate interactional space also seems to be based on kinship, encompassing the nuclear family in the shared residence, but also the frequently nominated grandparents. Since there is no immediate correspondence between kinship and residential proximity beyond the nuclear family, with many grandparents not living in the immediate neighborhood, this results in geographically broader networks. This kinship-based aspect of children’s legitimate interactional space is supplemented by institutional structures, facilitating access to individuals from broader areas, while restricting children from freely moving in their neighborhood. Most children of Bad Nauheim report to own a bicycle or a scooter, allowing them to independently cover a larger distance, helping them to maintain these broader networks.

Children of the Nseh seem to experience a continuous social context, as described for comparable settings (Weisner, 1984; Lancy, 2015), moving in the same social group throughout their childhood, thus knowing most of their attachment ties from their earliest days. The pattern described by the sample of Bad Nauheim, however, displays the social expansion commonly described in the attachment literature of middle childhood (Raikes and Thompson, 2005). In the setting of Bad Nauheim, most ties to peers are marked by a lower degree of consistency, originating from later developmental stages. However, there is also a considerable amount of peer relationships that are characterized by long-term stability, possibly contributing to the importance of same-aged peers as attachment figures in this sample. Generally, our data highlights that relationship stability needs further investigations. As of yet, it has been assumed that stability only applies to adult, parental attachment figures, leading to the exclusion of peers from further research. Since our data contradicts these theoretical reflections, future empirical research needs to address how relationship stability emerges based on ecocultural factors in order to understand to whom it applies in a specific setting and how it contributes to the selection of attachment figures.

Overall, we conclude that children in both settings rely on a network of attachment figures, with the exact size and composition of this array adapted to the context-specific care system and concepts of interconnectedness and the ecological features of each setting.

### The Adaptiveness of Functional Patterns Across Contexts

Concerning the functionality of their attachment ties in establishing a feeling of security and comfort, the children from both samples distinguished between various domains of security that they allocated to different attachment figures, highlighting the distribution of responsibilities across the network. While the community has already been identified as a source of security for relational contexts (Nsamenang, 1992, 1994; Keller, 2016), this data demonstrates the cooperative nature of attachment during middle childhood in *both* settings, with the functional impact of the entire network exceeding the impact of individual ties and a comprehensive feeling of security only established by a collective, again demonstrating the importance of an exhaustive assessment of all relevant individuals.

Considering these domains of security in both contexts, two different patterns emerge at first glance, reflecting context-specific themes and conditions that vastly differ across settings. Children of the Nseh talk about a world shaped by critical infrastructure and short supply with food. They have a responsible role as an active part of the workforce in the compound and at the farm, living in a hierarchical clan kept together by kinship ties. In their developmental environment, emotional expressions are only legitimate toward peers, being raised in a context of early self-regulation. Children of Bad Nauheim describe a world shaped by educational challenges and a (growingly) diverse array of social settings in which close connections to the nuclear family are supplemented by well-attuned ties to peers. While the setting offers physical stability overall, the continuous expansion and introduction to new settings and individuals amount to confrontations with socially challenging situations.

However, when acknowledging the interrelatedness of general developmental themes in middle childhood across developmental contexts (Weisner, 1984), avoiding the “ethnographic dazzle” (Fox, 1970, p. 12), it becomes apparent that the functional patterns can also be understood as children’s context-specific reflections on the same developmental challenges.

Firstly, middle childhood is generally a period of increasing agency, responsibilities and new contexts (Weisner, 1984; Ndze, 2008). Thus, superordinate individuals who continue to offer resources and support in these new challenges also provide a feeling of security. For children in the context of the Nseh, these new and increasing responsibilities concern their contributions at the farm and in the compound. Thus, individuals providing them with assistance in everyday life constitute important sources of security. As they also become responsible members of the care system, they reference their own responsibility for younger peers. For children of Bad Nauheim, these new responsibilities mostly concern the context of school (cf. LeVine, 1974, 1988). As a result, individuals providing support and orientation concerning children’s education constitute important sources of security, mostly applying to superordinate older peers and adults. Since they are also confronted with the expectancy of emotional self-regulation, they value those individuals who continue to guide them toward independence in social situations that are (as yet) emotionally challenging for them.

Secondly, children now have to independently care for their relationships, especially those to peers, and establish proximity and “shared intimacy” in these ties between equals (Weisner, 1984, p. 348). Thus, well-coordinated, functioning relationships to peers in which this developmental task has already been accomplished constitute another important source of stability and security. Children in Bad Nauheim talk about harmonious ties to peers that are well attuned, thus not depending on external, possibly adult regulation. Since social ties are less influenced by age-based hierarchical concepts than the Nseh, children of Bad Nauheim also describe a small group of relationships to adults as such ties of emotional attunement between equals. While children of the Nseh are broader in their descriptions, possibly resulting from cultural norms that restrict overt emotionality, they nonetheless also highlight the closeness and the affective relevance of their attachment ties, however, restricting this affection to relationships with same-aged peers. The limited importance of affection in relationships to superordinate attachment figures in the setting of the Nseh could result from the distribution of responsibilities after the first years of indulgent parental care, with peers now assuming affectional care while superordinate individuals retreat to a physical provision. However, it is also possible that these patterns result from interactional norms restricting children from expressing affective ties toward adults, especially when talking to an adult researcher, with aged-based norms and resulting social desirability shaping their responses (Becke et al., unpublished). Yet, it is important to consider that nutrition as the main functionality of attachment ties to adults does not only provide physical security, but can also encompass emotional comfort, with these ties possibly catering to children’s affective needs by indirect means (Carsten, 1995; Locher et al., 2005; Kaufmann, 2010).

Beyond these fundamental developmental themes, the children from both samples reflect on physical security, again demonstrating the adaptiveness of their functional patterns. Children of Bad Nauheim reference physical distress only in a small minority of attachment ties and mostly reference major incidents, possibly not experiencing their context as a continuous physical threat due to a stable infrastructure and high living standards (cf. LeVine, 1974, 1988). For children of the Nseh, however, nutrition, i.e., their physical integrity, constitutes their main source of (in-)security, living in an environment shaped by economic instability and a constantly perceived food shortage.

Lastly, children in both contexts confirm the importance of consistency, a relationship quality commonly understood as a basic requirement for the emergence of attachment ties and the feeling of security (Ainsworth, 1989; Mayseless, 2005). Children of Bad Nauheim directly describe the duration or range of a tie, referencing not only relationships with adults with kin ties, but also with peers they met in institutional settings who provide consistency, especially in transitional periods of institutional changes, further explaining the unexpected relevance of same-aged peers in the sample of Bad Nauheim. In the context of the Nseh, the importance of consistency is only indirectly expressed, using references of kinship as a context-specific marker for relationship stability. This social cohesion of the clan ties individuals permanently together, providing children with a continuous developmental setting.

Concerning the influence of age on perceived functionality, only the Cameroonian sample demonstrates a statistically significant differentiation, possibly resulting from stronger age-based hierarchical norms and resource allocations.

### Limitations and Future Research

Concerning our methodological strategy, future research will need to supplement these results with observational data and possibly also with reflections from attachment figures themselves since our data only relies on the children’s own account. Observational data will also help to further assess how different perceived functionalities possibly translate into differing interactional patterns and to further confirm the ecological validity of our approach. To investigate the influence of age-based interactional norms possibly shaping children’s responses, specifically in the setting of the Nseh, replication studies would need to employ same-aged peers as instructors and interviewers, sharing the children’s age group and cultural background. Including children in the coding process could possibly help to further bridge the gap of understanding between investigated children and researchers.

Since we only focused on middle childhood, it will be also necessary to assess the children’s own perspective on their attachment ties in other developmental stages in order to analyze how children’s attachment networks and the perceived functionalities of their ties change with the transformations of their developmental paths, especially focusing on transitional periods.

Concerning our approach of investigating the adaptiveness of children’s psychosocial development and the applicability of the observed patterns, it is important to consider that we only investigated two settings. While they were selected as examples for extreme sociodemographic settings and cultural models, we mostly focused on individual processes of adaption in the distinctive settings. Thus, it will be vital for future research to assess attachment patterns from several settings with a comparable sociodemographic setup in order to investigate the abstract impact of the fundamental cultural model on attachment during middle childhood beyond the individual context. Since this study only constitutes a first exploratory approach to link patterns of attachment during middle childhood with contextual features, future research will also need to consider the influence of other ecocultural characteristics of the multidimensional settings.

Concerning the data analysis, only a broad statistical analysis of individual dimensions could be conducted due to the exploratory nature of our study and the sample size. Larger samples in future research will help to include a more exact analysis and to also exclude that cultural differences only emerge based on possible sampling biases (van de Vijver and Leung, 2000).

On a conceptual level, there are still major limitations that also need to be addressed, especially concerning the definition of an ‘attachment figure’ with our broad assessment of anyone sustaining a feeling of safety possibly resulting in larger networks that include non-parental individuals. Data across studies can only be compared if a consistent understanding of the functionality and necessary range of an attachment figure has been established. Additionally, children of both investigated contexts will need to further differentiate attachment from other relationship types, e.g., companionship to substantiate that the ties assessed in this study actually reflect the children’s attachment network and not their general social environment.

## General Conclusion

Overall, by comparing children’s responses across both contexts, our study demonstrates that children’s attachment networks during middle childhood are shaped by features of their distinct developmental environment that influence the socio-structural allocation and the functionality of these ties. Thus, our data supports previous reflections on the adaptiveness of attachment to the ecocultural setting. However, general challenges of this distinct developmental stage also impact children’s attachment networks, resulting in structural and functional similarities across contexts; a pattern that only becomes apparent when contrasting two extreme settings.

From the children’s perspective in both contexts, attachment ties are more complex than the mother–child–dyads used in most previous approaches to study attachment during middle childhood. We conclude that a broad and exhaustive approach to attachment is important across developmental settings since network members seem to collectively establish and maintain children’s feelings of security across context-specific domains of security. Especially (same-aged) peers deserve closer research attention in the future, even in Western contexts, since children identify them as relevant for their feeling of security and since they describe that even a considerable amount of peer relationships can be marked by long-term stability.

Generally, an exploratory and transdisciplinary perspective on attachment investigating the distinct setting and focusing the children’s own perception generally needs to be considered as a possible addition to attachment research to develop an adequate understanding of children’s development across cultural boundaries.

## Data Availability

The raw data supporting the conclusions of this manuscript will be made available by the authors, without undue reservation, to any qualified researcher.

## Author Contributions

SDB conceived and planned the design of the study, conducted the recruitment and the data collection in both samples, and performed the data analysis and interpretation. SDB supervised the project, supported the statistical analysis, and discussed the interpretation of the results. SDB wrote the first draft of the manuscript. Both authors contributed to manuscript revision, read and approved the submitted version.

## Conflict of Interest Statement

The authors declare that the research was conducted in the absence of any commercial or financial relationships that could be construed as a potential conflict of interest.

## Footnotes

1 Pretests demonstrated that asking children to provide exact ages for their attachment figures did not generate reliable data in both settings. We thus simplified the categories, using cut-off lines that children can easily reconstruct based on external circumstances in the German setting of Bad Nauheim, while employing indigenous categories in the Cameroonian setting of Nseh.

2 Again, asking children to state an exact point of time proved not to generate reliable data in pretests in both settings.

3 Since we follow an exploratory approach in investigating children’s attachment networks with a limited sample size, we decided to translate our data into visual representations to focus on preliminary results and trends and make general patterns easily accessible.

4 Individuals with an exclusive institutional tie were excluded from the analysis since children were not able to identify their residential category.

5 For the sample of the Nseh, children stated perceived functionality for 134 individuals.
