# Supplementary material for: Comparing Attachment Networks During Middle Childhood in Two Contrasting Cultural Contexts
Source: Front Psychol. 2018 Jul 17;9:1201. doi: 10.3389/fpsyg.2018.01201 (PMC6057239; doi:10.3389/fpsyg.2018.01201)
Supplement: Supplementary file 1 [file Table_1.pdf]

Supplementary Material

Comparing Attachment Networks during Middle Childhood in Two Contrasting Cultural Contexts

Sophia Daphne Becke*, Stephan Bongard

*** Correspondence:** Sophia Daphne Becke: becke@psych.uni-frankfurt.de

Supplementary Table 1. Distribution of age categories in both settings

	Cameroonian sample of the Nseh *N* = 171		German sample of Bad Nauheim *N* = 108	
Age category	*N*	%	*N*	%
Younger peers	30	17.6	3	2.8
Same-aged peers	54	31.6	38	35.2
Older peers	24	14.0	7	6.5
Adults	63	36.8	60	55.5

Note. Percentages relate to the individual setting

Supplementary Table 2. Distribution of gender categories in both settings

	Cameroonian sample of the Nseh *N* = 171				German sample of Bad Nauheim *N* = 108			
	Female participants		Male participants		Female participants		Male participants	
Gender	*N*	%	*N*	%	*N*	%	*N*	%
Female attachment figures	58	59.8	36	48.6	37	71.2	25	44.6
Male attachment figures	39	40.2	38	51.4	15	28.8	31	55.4

Note. Percentages relate to each gender of participants in each setting.

Supplementary Table 3. Distribution of residential categories in both settings

	Cameroonian sample of the Nseh *N* = 171		German sample of Bad Nauheim *N* = 103	
Residential category	*N*	%	*N*	%
Shared residence	61	35.7	38	36.9
Shared neighborhood	59	34.5	10	9.7
Shared town	28	16.4	32	31.1
Out of town	23	13.4	22	21.3
Abroad	-	-	1	1.0

Notes. Percentages relate to the individual setting
Individuals with an exclusive institutional tie were excluded from the analysis since children were not able to identify their residential category.

Supplementary Table 4. Distribution of duration categories in both settings

	Cameroonian sample of the Nseh *N* = 171		German sample of Bad Nauheim *N* = 108	
Duration category	*N*	%	*N*	%
Since birth	162	94.7	73	67.6
Since early childhood	7	4.1	17	15.7
Since middle childhood	-	-	17	15.7
Less than 1 year	2	1.2	1	0.9

Note. Percentages relate to the individual setting

Supplementary Table 5. Distribution of categories of perceived functionality across age categories for the German sample of Bad Nauheim

	Younger peers *n* = 3		Same-aged peers *n* = 38		Older peers *n* = 7		Adults *n* = 60		Total *N* = 108	
Functional category	*N*	%	*N*	%	*N*	%	*N*	%	*N*	%
Socio-emotional assistance	-	-	14	36.8	2	28.6	17	28.3	33	30.5
Consistency	1	33.3	8	21.1	2	28.6	19	31.7	30	27.8
Harmonization	1	33.3	11	28.9	-		10	16.7	22	20.4
Education	-	-	2	5.3	3	42.8	9	15.0	14	13.0
Instrumental assistance in distress	1	33.3	3	7.9	-		5	8.3	9	8.3

Note. Percentages relate to the individual age groups

Supplementary Table 6. Distribution of categories of perceived functionality across age categories for the Cameroonian sample of the Nseh

	Younger peers *n* = 24		Same-aged peers *n* = 44		Older peers *n* = 21		Adults *n* = 45		Total *N* = 134	
Functional category	*N*	%	*N*	%	*N*	%	*N*	%	*N*	%
Nutritional care	4	16.7	9	20.5	11	52.4	22	48.9	46	34.3
Kinship	11	45.8	10	22.7	-	-	13	28.9	34	25.4
Everyday instrumental assistance	3	12.5	10	22.7	8	38.1	8	17.8	29	21.6
Affection	6	25.0	15	34.1	2	9.5	2	4.4	25	18.7

Notes. Percentages relate to the individual age groups
For the Cameroonian sample, children stated perceived functionality for 134 individuals.
